# Translocations associated with colour-sidedness are common in northern Swedish cattle breeds

**DOI:** 10.1186/s41065-025-00481-w

**Published:** 2025-07-07

**Authors:** Julia Hinken, Tytti Vanhala, Marta Gòdia, Martin Johnsson, Anna M. Johansson

**Affiliations:** 1 Department of Animal Biosciences, Swedish University of Agricultural Sciences, Box 7023, 750 07 Uppsala, Sweden; 2 Animal Breeding and Genomics, Wageningen University and Research, Wageningen, Netherlands

**Keywords:** Gonadal hypoplasia, Swedish Mountain cattle, Structural variants

## Abstract

The Swedish Mountain cattle and four other native Swedish cattle breeds show the phenotype of colour-sidedness. The causes of this phenotype are either a translocation and duplication from chromosome 6 to chromosome 29 (known as Cs_29_) including the *KIT* gene, or an additional translocation allele where part of Cs_29_ has been translocated back to chromosome 6 (Cs_6_). Besides the colour-sidedness, the Cs_29_ translocation has been associated with gonadal hypoplasia which can cause fertility problems. Despite breeding efforts during more than 80 years to reduce the prevalence of gonadal hypoplasia, there are still individuals with gonadal hypoplasia. We genotyped the Cs_29_ and Cs_6_ alleles in Swedish Mountain cattle and five other Swedish native breeds. Whole-genome sequence data of 30 cattle were analysed for the translocations, and 115 DNA samples were analysed using multiplex PCR. The current allele frequency of the Cs_29_ allele in Swedish mountain cattle was estimated to be 44%. The results indicate that the Cs_29_ translocation is also present in the four other native Swedish cattle breeds with colour-sidedness, and the Cs_6_ translocation in at least three of them.

**Supplementary Information:**

The online version contains supplementary material available at 10.1186/s41065-025-00481-w.

## Background

The Swedish Mountain cattle (Fjällko) is a native breed which is known to be healthy and robust and once played an important role for milk production in northern Sweden [1, 2]. In 2021 the breed consisted of only 7,802 breeding individuals: 2,032 breeding males and 5,770 breeding females [3]. Despite the small population size, the breed shows a relatively high genetic diversity [4]. Furthermore, Swedish Mountain cattle show a higher frequency of the β-casein variants A^1^ and B as well as the κ-casein variant B compared to conventional milk production breeds [5].

The breed was established in the 1880 s and in 1893 breeders came up with a breed standard [6] that included a mainly white coat colour with red or black dots on their flanks and coloured ears and polledness [2, 3, 6]. With selection based on the white coat colour, gonadal hypoplasia increased in the breed [7], because of an unfavourable genetic relationship with colour-sidedness, which was not known at the time. Gonadal hypoplasia is a disease in which one or both gonads, ovaries in cows and testicles in bulls, are small and underdeveloped. In Swedish Mountain cattle the affected ovary or testicle is predominantly the left one, but cases with right sided or double-sided hypoplasia can be found [8, 9].

The origin of gonadal hypoplasia in Swedish Mountain cattle is suspected in Jämtland in 1900 [7]. In the following years fertility problems as well as the number of affected animals increased, and researchers later found out that hypoplasia was an inherited disorder [8]. Therefore, the Swedish Mountain cattle breeding association decided in 1937 that hypoplasia affected bulls could no longer be added to the herd book, to reduce the occurrence of gonadal hypoplasia in the breeding population [8]. From 1943 onwards breeding bulls must have an unaffected mother and since 1950 an unaffected maternal grandmother [8]. This phenotypic control program is still in use [10].

The genetic mechanism responsible for colour-sidedness is a translocation of ~ 500 kbp from chromosome 6 to chromosome 29 (known as the Cs_29_ allele), or another translocation, called Cs_6_, which involves a translocation back to chromosome 6 of a region from Cs_29_ [11]. The translocated segment includes the *KIT* gene which is known to encode a type III receptor protein of the tyrosine kinase family. The KIT protein is needed for survival, proliferation and migration of melanocyte precursors and primordial germ cells during embryogenesis as well as regular development of hematopoietic stem cells [12–15]. In fact, SNP variants in the *KIT* gene were found to be associated with fertility and be identified as one of the several causes of male infertility in humans [16]. Besides their role in reproduction, the *KIT* gene has also been associated with white pigmentation and spotting in several mammals, including pigs [17]. In addition to colour-sidedness, the Cs_29_ allele has been associated with gonadal hypoplasia in Northern Finncattle, making it a plausible candidate variant for gonadal hypoplasia in Swedish Mountain cattle [9].

In this study, we aimed to investigate the occurrence of the Cs_29_ and Cs_6_ translocations in Swedish Mountain Cattle and other local Swedish cattle breeds. We used whole-genome sequence data from 30 cattle of different Swedish local breeds, as well as multiplex breakpoint PCR genotyping of 55 individuals born between 1976 and 2015 and 80 of contemporary cattle sampled in 2023–2024. These animals belong to Swedish Mountain cattle, three closely related breeds Fjällnära, Bohus Polled (Bohuskulla) and Swedish Polled (Svensk Kullig Boskap), as well as the Väneko breed which is not as closely related to the others but has colour-sided animals.

## Material and methods

### Translocation genotyping by sequence depth of coverage

Whole-genome Illumina sequence data from 30 animals belonging to Swedish native breeds was previously generated by [18]. These animals were sequenced on the Illumina NovaSeq 6000 platform with mapped genome coverages ranging from 15 to 42X with an average of 26X. The objective of this analysis was to use depth of coverage as a proxy to genotype the translocation alleles. The breed distribution from the 30 Swedish cattle from different local breeds is shown in Table 1.

**Table 1:** Number (N) of whole-genome sequenced individuals from the different Swedish cattle breeds

N	Breed
9	Swedish Red Polled (Rödkulla)
7	Swedish Mountain (Fjäll)
5	Väne
4	Fjällnära
3	Bohus Polled (Bohuskulla)
2	Ringamåla

We performed alignment and the first step of variant calling with GATK using the Sarek workflow version 2.7.1 [19, 20]. Briefly, sequence reads were aligned to the cattle reference genome ARS-UCD1.2 using bwa mem [21]. Picard (2.27.1) (http://broadinstitute.github.io/picard/) was then used to mark duplicate reads. Following, we used BEDTools (version 2.30.0) [22] to calculate the depth of coverage in 10 kbp windows along the cattle genome. The depth of coverage was standardized by dividing the depth of coverage in each window by the median depth of all windows in the genome. We plotted the depth of coverage in two 1.5 Mbp regions of interest defined by converting the regions of interest from [9] to the ARS-UCD1.2 assembly with UCSC LiftOver (https://genome.ucsc.edu/cgi-bin/hgLiftOver) using default settings. This allowed us to detect elevated depth of coverage in the regions affected by the translocations Cs_29_ and Cs_6_. To distinguish genotypes, we visually compared the standardized depth of coverage in regions affected by the translocation to the expected depth from different genotypes. Since the expected elevation of coverage is 1.5 times in heterozygotes and 2 times in homozygotes, we plotted horizontal lines corresponding to 1.5 times and 2 times the median depth, respectively.

### Samples for PCR genotyping

We analysed DNA samples from 59 (of which 55 had clear results that we could use) individuals from native Swedish cattle breeds born between 1976 and 2015 (Table 2), and 80 of contemporary animals sampled in 2023–2024. The gonadal hypoplasia status of most animals was unknown. The DNA samples were already collected in the past for previous research. The DNA had been extracted from blood and sperm samples and was stored at −20 °C. Quality control was done with NanoDrop (Thermo Fisher Scientific Inc), and 21 samples with low concentrations were also analysed with the Qubit dsDNA BR assay kit (Invitrogen).

**Table 2:** Number of samples from the different Swedish cattle breeds with successful PCR genotyping. (Within brackets are the number of samples that were collected 2023–2024.)

N	Breed
80 (60)	Swedish Mountain (Fjäll)
35 (20)	Fjällnära
8	Swedish Polled (Svensk Kullig Boskap)
7	Bohus Polled (Bohuskulla)
5	Väne

### Breakpoint primers

Six breakpoint PCR primer pairs were developed by [11] and [9]. They span breakpoints of the translocation and fusion points on chromosome 6 and chromosome 29. The α-D, A-E and C-β primer pairs detect the Cs_29_ allele which is responsible for the colour-sidedness and gonadal hypoplasia. The γ-B primer pair detects the Cs_6_ allele also known to cause colour-sidedness. The α-β and α-β_2 primer pairs detect the wild type allele on chromosome 29. We used online BLAST searches through the Ensembl genome browser [23] to confirm that the primers aligned in the expected regions on chromosomes 6 and 29 in the ARS-UCD1.2 reference genome [24]. We then used the UCSC in-silico PCR tool [25] to verify no other segments were predicted to amplify.

### Sanger sequencing

Four individuals with whole-genome sequence data were also Sanger sequenced with the BigDye Direct Cycle Sequencing Kit (Applied Biosystems, Life Technologies) to validate the six different primer pairs using the Sanger sequencing technique [26]. The primers were tailed with the M13 universal primer sequences. The sequencing was performed according to the kit manual with the exception of the annealing temperature for PCR being 58 °C instead of 62 °C. After amplification, the samples were cleaned with the BigDye Xterminator Purification Kit (Applied Biosystems, Life Technologies) according to the protocol to remove unincorporated terminators and salts. The purified samples were then run in the capillary electrophoresis (3500xL Genetic Analyzer, [27]. The results of the sequencing were aligned by using MEGA11 [28] and searched for its position against the cattle genome by using the Ensembl BLAST web tool [23].

### PCR

The six primer pairs (α-D, A-E, C-β, γ-B, α-β and α-β_2) were tested on the same four individuals that were used in Sanger sequencing. After testing the primer pairs individually, a multiplex of three informative primer pairs, A-E, γ-B and α-β, could be constructed.

All the amplifications were done using HotStarTaq Plus Master Mix kit (Qiagen). The standard protocol of the kit was followed with the exception of using a final volume of 10 µl and 10 ng of template DNA. For single primer pair runs, we used 0.5 µM of each forward and reverse primer. For the multiplex, 0.25 µM of each forward and reverse primer for A-E and α-β, and 0.4 µM for γ-B was used. The PCR reactions were run on the ProFlex PCR system (Applied Biosystems, Life Technologies). The initial denaturation was done at 95 °C for 5 min, followed by 35 cycles at 94 °C for 30 s, 58 °C for 1 min and 72 °C for 1 min. The final extension was done at 72 °C for 10 min.

PCR products were visualized with the Agilent 4200 TapeStation system using the High Sensitivity D1000 kit (Agilent Technologies Inc.). The A-E primer pair with a product of 318 bp is an indicator of the Cs_29_ translocation. The α-β primer pair product of 394 bp marks the wildtype allele, and the γ-B product of 525 bp is an indicator for the Cs_6_ translocation.

### Genotype and allele frequencies

We calculated the observed genotype and allele frequencies for each breed. For Cs_29_ we report allele frequencies. Because the breakpoint PCR cannot distinguish Cs_6_ homozygotes from heterozygotes (acting as a dominant marker), we report only frequencies of presence of Cs_6_. The 95% confidence intervals were calculated using a binomial test in R [29]. We tested significance of difference in genotype frequencies using the fisher exact test in R [29].

## Results

### Detection of the translocation alleles from whole-genome sequence data

**Fig. 1:**
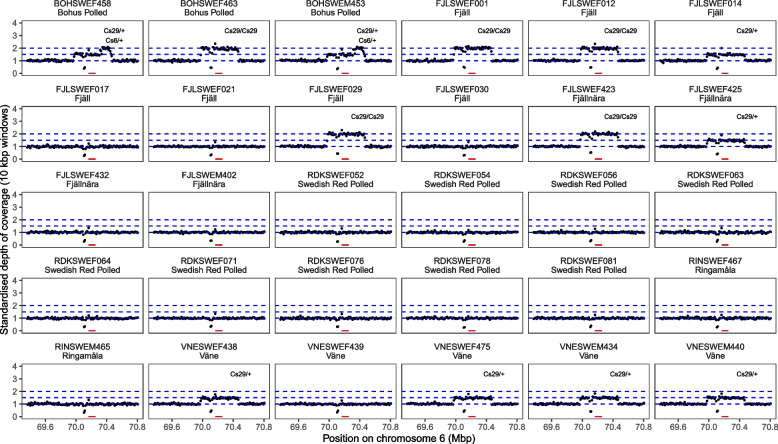
Standardized depth of coverage within and surrounding the regions duplicated by Cs_29_ and Cs_6_ on chromosome 6 for each of the samples with whole genome sequence data. The red rectangle represents the location of the *KIT* gene (ENSBTAG00000002699). The blue dashed lines show the 1x, 1.5 × and 2 × times the median depth of coverage in the region. The inset label gives the inferred translocation genotypes for Cs_29_ and Cs_6_

**Fig. 2:**
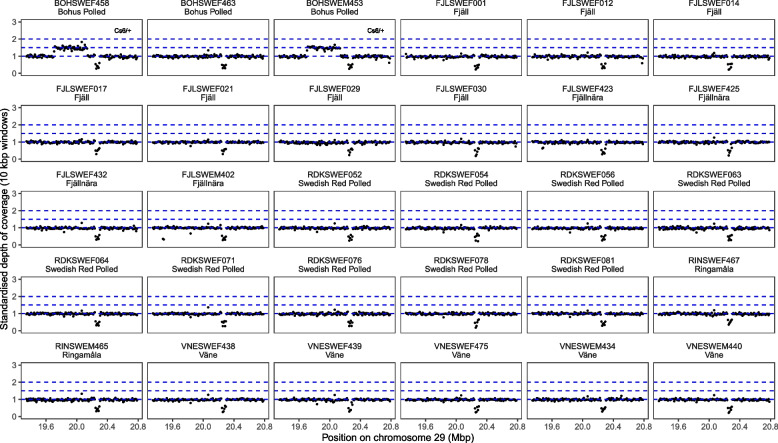
Standardized of coverage in and around the region affected by Cs_6_ on chromosome 29. The blue dashed lines show the 1x, 1.5 × and 2 × times the median depth of coverage in the region. The inset label gives the inferred translocation genotype for Cs_6_

We detected the Cs29 translocation in sequenced animals of the Swedish Mountain cattle, Fjällnära, Väne cattle and Bohus Polled breeds. The depth of coverage in the region around the regions affected by Cs_29_ and Cs_6_ on chromosome 6, and their inferred genotypes, was clearly identified (Fig. 1). There were five homozygotes and eight heterozygotes. The translocation was not detected in the Swedish Red Polled and Ringamåla breed, which are not colour-sided. In addition, two individuals of the Bohus Polled breed were heterozygous for the Cs_6_ translocation, also indicated by elevated coverage in the region affected by Cs_6_ on chromosome 29 (Fig. 2).

### Detection of translocations by multiplex breakpoint PCR

We detected the Cs_29_ allele in all breeds tested by multiplex breakpoint PCR: Swedish Mountain cattle, Fjällnära, Bohus Polled, Väne cattle and Swedish Polled. We also detected the Cs_6_ allele in all breeds except in Väne cattle. The genotypes are shown in Additional file 1. Six animals that were whole-genome sequenced were also genotyped with breakpoint PCR, and their genotypes agreed.

In the animals born 1976–2015, allele frequencies of the Cs_29_ translocation ranged between 0.22 in Swedish Polled and up to 0.57 in Swedish Mountain cattle (Table 3). In contemporary animals sampled 2023–2024, allele frequencies were 0.44 for Swedish Mountain cattle and 0.15 for Fjällnära, which is a decrease compared to animals sampled earlier. As can be seen in Table 3 the confidence intervals of allele frequencies for Cs_29_ do overlap indicating that the differences in frequency between the year cohorts are not significant. Also the result of the fisher exact test of the genotype frequencies showed that there were no significant differences between the time periods (*p* = 0.229 and 0.155 respectively for the breeds). For the Cs_6_ allele no distinction between hetero- and homozygous individuals was possible, and therefore the frequency of Cs_6_ positive (heterozygous or homozygous) animals are reported.

**Table 3:** Allele frequencies of the Cs29 translocation in the different breeds (results from PCR typing and WGS data are added)

Breed	Allele frequency Cs_29_ (95% confidence interval)	Cs_6_ positive genotype frequency (95% confidence interval)
Swedish Mountain (Fjäll) (*n* = 25), sampled in 1990 s and 2004–2015	0.56 (0.41–0.70)	0.40 (0.21–0.61)
Swedish Mountain (Fjäll) (*n* = 60), sampled 2023–2024	0.44 (0.35–0.54)	0.35 (0.23–0.48)
Fjällnära (*n* = 16), sampled in 1990s	0.28 (0.14–0.47)	0.31 (0.11–0.59)
Fjällnära (*n* = 20), sampled 2023–2024	0.15 (0.06–0.30)	0.45 (0.23–0.68)
Bohus Polled (*n* = 9), sampled in 1990s	0.67 (0.41–0.87)	0.33 (0.07–0.70)
Swedish Polled (*n* = 8)	0.31 (0.11–0.59)	0.25 (0.03–0.65)
Väne (*n* = 10), sampled in 1990s	0.40 (0.19–0.64)	0 (0–0.31)

## Discussion

The aim of this research was to investigate the occurrence of translocation alleles associated with colour-sidedness and gonadal hypoplasia in several native Swedish cattle breeds. The results showed that the Cs_29_ is common both in Swedish Mountain cattle and in Fjällnära, Bohus Polled, Väne cattle and Swedish Polled. This might be related to the fact that all breeds show the colour-sidedness phenotype and most of them are related to the Swedish Mountain cattle. We also found the Cs_6_ translocation was found present in all the above-mentioned breeds except Väne cattle. In contemporary animals sampled in 2023–2024, the frequency of the Cs_29_ allele was high in Swedish Mountain cattle (44%), albeit somewhat lower than in the animals born 1976–2015 (56%). In Fjällnära the frequency of Cs_29_ was also lower with 15% in contemporary animals compared to 28%, although these differences were not significant.

### Comparison to previous results

Durkin et al. [11], who first identified the translocations, investigated colour-sided animals from eight breeds, including two Swedish mountain cattle, where they found both Cs_29_ and Cs_6_. Venhoranta et al. [9] found an allele frequency of Cs_29_ of 60% in Northern Finncattle, and their sample also included a few Swedish Mountain cattle. They also found a frequency of 61% Cs_6_ positive animals (heterozygous and homozygous). This suggests that both translocations are more common in Northern Finncattle than in Swedish Mountain cattle, but that the difference is bigger for Cs_6_.

### Limitations

There are several limitations to keep in mind when interpreting our results. First, the sample sizes for some breeds are relatively small, especially for Bohus Polled, Swedish Polled and Väneko, and mostly from individuals born before 2000. Second, the samples were not taken at random, and may not be representable for the whole population. Our estimates from the older samples and smaller breeds in particular should therefore be interpreted cautiously. The contemporary samples from Swedish mountain cattle 2023–2024, however, are in total 60 samples and are born in eight different herds, and likely give a fairly good representation of the diversity of the breed at present. Because the hypoplasia status of most of the samples was not known, we cannot estimate the phenotypic prevalence or the penetrance. To know the current incidence of gonadal hypoplasia and test the previously reported association to Cs_29_, samples of clinically examined animals need to be collected.

### Implications for gonadal hypoplasia

In addition to the effect on colour-sidedness, the Cs_29_ allele but not Cs_6_ has been associated with gonadal hypoplasia in Northern Finncattle [9]. If these results hold up in Swedish Mountain cattle, genotyping and selection against hypoplasia would be possible. Our results show that the Cs_29_ translocation allele is common, despite a phenotypic control program aimed at keeping hypoplastic bulls out of breeding [8]. The incidence of gonadal hypoplasia was measured in the 1930 s to 1950 s, when the hypoplasia problem was noticed and the control program instituted [7] reported frequencies for gonadal hypoplasia between 26 and 30% in 1935, 23% in 1937 and 8% in 1942. Previous work [8] found incidences of 18% for cows born until 1936, for cows born between 1937 and 1939 it was 15%, for cows born between 1940 and 1945 it was 11% and for cows born between 1946 and 1948 it was 9% [30] estimated the incidence of gonadal hypoplasia in organs from cows slaughtered between 1950 and 1952. The incidence of total hypoplasia was 5.2% to 11.1% in different counties and the incidence of partial hypoplasia was 3.8% to 8.0% in different counties. Thus, the three studies reported a declining incidence for gonadal hypoplasia over time. Several factors may contribute to an increase in Cs_29_ even in face of the control program. One of them is the reduction of the Swedish Mountain cattle breeding population. Another contributing factor could have been the use of artificial insemination bulls who carry Cs_29_. The allele could be found in seven breeding bulls born in the twenty-first century included in this study, including two bulls homozygous for Cs_29_. In the samples collected in 2023–2024, the frequency was lower, indicating that the control program does work.

In order to improve the accuracy over the phenotypic control program, the multiplex breakpoint PCR could be developed into a DNA test for gonadal hypoplasia. The breeding organisation already offers the possibility of DNA analysis for milk casein, so no extra DNA collection would be necessary, just an additional DNA test [31]. The test for Cs_29_ would give a clear marker genotype for the hypoplasia and make selection more accurate. However, there is still the question on what to do with affected individuals. Excluding all carriers from breeding would narrow the genetic material and increase the risk of inbreeding. It seems more reasonable to select against homozygous bulls, by either excluding them from breeding or limiting their use. Further, if cows could be genotyped, the DNA test could be used to avoid carrier–carrier matings. Before being implemented, these interventions will need to be evaluated for their consequences for genetic diversity and cost to the breeding program.

## Conclusion

Translocations associated with colour-sidedness are common in Swedish Mountain cattle and as present in several other native Swedish cattle breeds (Bohus Polled, Swedish Polled, Väneko and Fjällnära). If the previously reported association between one of these translocations and gonadal hypoplasia hold up in Swedish Mountain cattle, genotyping and selection against hypoplasia might be possible.

## Supplementary Information

Additional file 1. Translocation genotypes from multiplex breakpoint PCR.

## Data Availability

Whole-genome sequence data used in this paper are available in the European Nucleotide Archive with project accession PRJEB60564. The genotypes from multiplex breakpoint PCR are contained in the supporting materials.
